# Supplementary material for: Sexual dimorphism in striatal dopaminergic responses promotes monogamy in social songbirds
Source: eLife. 2017 Aug 11;6:e25819. doi: 10.7554/eLife.25819 (PMC5584986; doi:10.7554/eLife.25819)
Supplement: Transparent reporting form [file elife-25819-transrepform.docx]

***eLife’s* transparent reporting form**

We encourage authors to provide detailed information *within their submission* to facilitate the interpretation and replication of experiments. If you have any questions, please contact us: [editorial@elifesciences.org](mailto:editorial@elifesciences.org).

**Sample-size estimation**

- You should state whether an appropriate sample size was computed when the study was being designed
- You should state the statistical method of sample size computation and any required assumptions
- If no explicit power analysis was used, you should describe how you decided what sample (replicate) size (number) to use

Please outline where this information can be found within the submission (e.g., page numbers or figure legends), or explain why this information doesn’t apply to your submission:

We used a sample size similar to Salimpoor et al (Nature Neuroscience 2011) PET study in humans about striatal dopaminergic activation during music stimulation, which had a similar experimental design to ours^1^ (page 6; also see Methods pages 16, 19). Comparable sample sizes were used in other recent PET studies in birds^2^, rats^3,4^ and humans^5^. We also followed the guidelines of the Andreasen et al. (1996) methods paper on sample size and statistical power in PET studies. This paper found that as sample size decreases, false negatives begin to appear, but there is no corresponding increase in false positives^6^ – and in the present work we report positive PET results, cross-validated by behavioral experiments.

**Replicates**

- You should report how often each experiment was performed
- You should include a definition of biological versus technical replication
- The data obtained should be provided and sufficient information should be provided to indicate the number of independent biological and/or technical replicates
- If you encountered any outliers, you should describe how these were handled
- Criteria for exclusion/inclusion of data should be clearly stated
- High-throughput sequence data should be uploaded before submission, with a private link for reviewers provided (these are available from both GEO and ArrayExpress)

Please outline where this information can be found within the submission (e.g., page numbers or figure legends), or explain why this information doesn’t apply to your submission:

We report both biological and technical replicates in the Methods section, namely ‘Experimental design’ (pages 13-14) and protocol for ‘Simultaneous PET on four zebra finches to measure dopamine released during auditory stimulation in awake unrestrained state’ (pages 16-18). One group had an outlier, as can be seen in Fig. 3 – Figure Supplement 1; it was not excluded, because the difference remained significant. Some PET data had to be excluded, for similar reasons as in the previously published work^1^, which is described in the section on ‘PET image preparation and statistical analysis’ (page 19). All our PET results were cross-validated by behavioral experiments, and the outcome of both for each group is presented in the neighboring figures (Fig. 3 & 4, 5 & 6, 7 & 8).

**Statistical reporting**

- Statistical analysis methods should be described and justified
- Raw data should be presented in figures whenever informative to do so (typically when N per group is less than 10)
- For each experiment, you should identify the statistical tests used, exact values of N, definitions of center, methods of multiple test correction, and dispersion and precision measures (e.g., mean, median, SD, SEM, confidence intervals; and, for the major substantive results, a measure of effect size (e.g., Pearson's r, Cohen's d)
- Report exact p-values wherever possible alongside the summary statistics and 95% confidence intervals. These should be reported for all key questions and not only when the p-value is less than 0.05.

We provide individual data for all experiments, as suggested for groups of N<10 (Fig. 3, 3S2, 4, 5S1, 6, 7, 8). All figure legends with data state the statistical tests used and N (Fig. 3-8); statistical tests are stated whenever p-value is reported in the text (pages 7-14). Exact p-values are shown for all experiments (Fig. 3-4, 6-8, 3S1, 3S2; Tables 1-4). Statistical analysis is described in the Methods section ‘PET image preparation and statistical analysis’ (pages 19-20).

Please outline where this information can be found within the submission (e.g., page numbers or figure legends), or explain why this information doesn’t apply to your submission:

(For large datasets, or papers with a very large number of statistical tests, you may upload a single table file with tests, Ns, etc., with reference to page numbers in the manuscript.)

**Additional data files (“source data”)**

- We encourage you to upload relevant additional data files, such as numerical data that are represented as a graph in a figure, or as a summary table
- Where provided, these should be in the most useful format, and they can be uploaded as “Source data” files linked to a main figure or table
- Include model definition files including the full list of parameters used
- Include code used for data analysis (e.g., R, MatLab)
- Avoid stating that data files are “available upon request”

Please indicate the figures or tables for which source data files have been provided:

N/A

1	Salimpoor, V. N., Benovoy, M., Larcher, K., Dagher, A. & Zatorre, R. J. Anatomically distinct dopamine release during anticipation and experience of peak emotion to music. *Nat Neurosci* **14**, 257-262, doi:nn.2726 [pii]

10.1038/nn.2726 (2011).

2	Marzluff, J. M., Miyaoka, R., Minoshima, S. & Cross, D. J. Brain imaging reveals neuronal circuitry underlying the crow's perception of human faces. *Proc Natl Acad Sci U S A* **109**, 15912-15917, doi:1206109109 [pii]

10.1073/pnas.1206109109 (2012).

3	Cocker, P. J., Dinelle, K., Kornelson, R., Sossi, V. & Winstanley, C. A. Irrational choice under uncertainty correlates with lower striatal D(2/3) receptor binding in rats. *J Neurosci* **32**, 15450-15457, doi:10.1523/JNEUROSCI.0626-12.2012 (2012).

4	Michaelides, M. *et al.* PET imaging predicts future body weight and cocaine preference. *Neuroimage* **59**, 1508-1513, doi:10.1016/j.neuroimage.2011.08.028 (2012).

5	Small, D. M., Jones-Gotman, M. & Dagher, A. Feeding-induced dopamine release in dorsal striatum correlates with meal pleasantness ratings in healthy human volunteers. *Neuroimage* **19**, 1709-1715 (2003).

6	Andreasen, N. C. *et al.* Sample size and statistical power in [15O]H2O studies of human cognition. *J Cereb Blood Flow Metab* **16**, 804-816, doi:10.1097/00004647-199609000-00005 (1996).
